# Depression and Catatonia: A Case of Neuropsychiatric Complications of Moyamoya Disease

**DOI:** 10.7759/cureus.3460

**Published:** 2018-10-16

**Authors:** Jonathan Lai, Abdurraoof Patel, Charlotte Dandurand, Peter Gooderham, Shaohua Lu

**Affiliations:** 1 Pathology, St. George's University School of Medicine , St George, GRD; 2 Internal Medicine, The Brooklyn Hospital Center, Affiliate of the Mount Sinai Hospital, New York, USA; 3 Neurosurgery, Vancouver General Hospital, University of British Columbia, Vancouver, CAN; 4 Psychiatry, Vancouver General Hospital, University of British Columbia, Vancouver, CAN

**Keywords:** mmd, moyamoya disease, rnf213 gene, depression, catatonia, stroke, benzodiazepine, internal carotid arteries, icas

## Abstract

Moyamoya disease (MMD) is a rare idiopathic cerebrovascular disease most common among the Asian population. Studies have shown that patients with MMD are at increased risk for developing psychiatric complications. We present a patient with hemorrhagic MMD (RNF213 gene mutation) who developed depression and catatonia over time following MMD-related strokes. While no guidelines exist for the management of such an uncommon scenario, it at least requires an interdepartmental approach. Our report highlights the medical complications of untreated MMD and its neuropsychiatric association with depression and catatonia.

## Introduction

Moyamoya disease (MMD) is a progressive steno-occlusive disease within the circle of Willis, particularly affecting the distal segments of the internal carotid arteries (ICAs). Reduced blood flow in these major vessels lead to the formation of compensatory collateral circulation [1]. The appearance of abnormally dilated collateral vessels on angiography is reflective of “something hazy, like a puff of cigarette smoke,” which, in Japanese, is called moyamoya [1]. The incidence of MMD has a bimodal distribution, with regard to age, as it peaks in two age groups: approximately 5 year olds, and people in their mid-40s [2]. Geographically, although MMD was initially reported in Japan due to a high incidence, many cases have also been reported around the world. The incidence of MMD in Japan is about three cases per 100,000, in Europe its incidence is about three cases per 1,000,000 (one tenth of that observed in Japan), and in America the incidence is 86 cases per 100,000 people [1,2]. In Japan, females are predominantly affected by this condition [2].

While the etiology of MMD is largely unknown, it has been reported to have genetic determinants associated with a number of underlying conditions [3]. Several genetic loci such as 3p24-p26, 6q25, 8q23, and 17q25 have been correlated with MMD with polymorphism on ringer protein 213 (RN213) associated with the worst prognosis [4]. However, the current model of the genetic determinism of MMD reports the polymorphism of the ring finger protein 213 (RNF213) on chromosome 17 being a major factor as it has the worst prognosis [4]. Moreover, the neuropsychiatric manifestations associated with MMD are unusual. Further research is needed to document the psychiatric manifestations, as well as stratify cognitive symptoms according to disease severity and management. Here in, we report a case of a 34-year-old Asian female with MMD who developed catatonia and depression. We discuss the salient neuropsychological features of MMD.

## Case presentation

**Figure 1:**
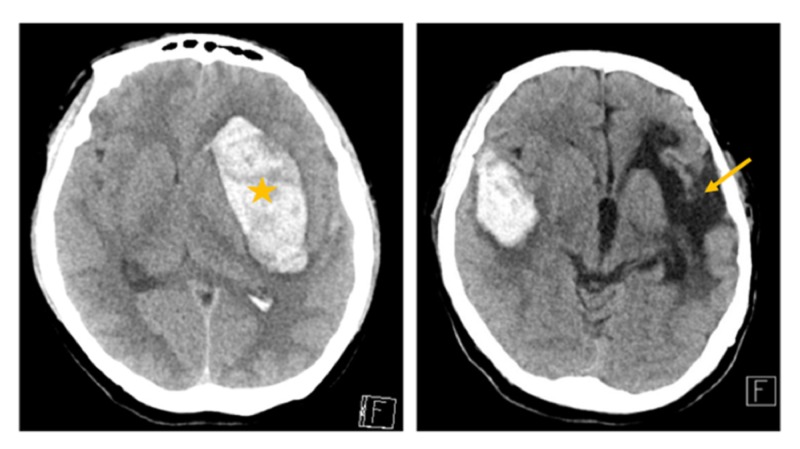
(Left) First hemorrhagic stroke on the left hemisphere (star). (Right) Right temporal hemorrhagic stroke with surrounding vasogenic edema with left-sided encephalomalacia from a previous hemorrhagic stroke (arrow).

A 34-year-old Chinese female with a previous medical history of interventricular hemorrhages was admitted for refractory migraines and changes in behavior. During the year, the patient’s family noted behavioral changes stating that she seemed depressed for at least three months, with fluctuating mood, decreased appetite, increased somnolence, and bizarre behavior. She also seemed to be less active and was not interested in her regular activities. She was brought to the hospital after she was found unresponsive, and a right temporal intracranial hemorrhage was detected (Figure 1).

**Figure 2:**
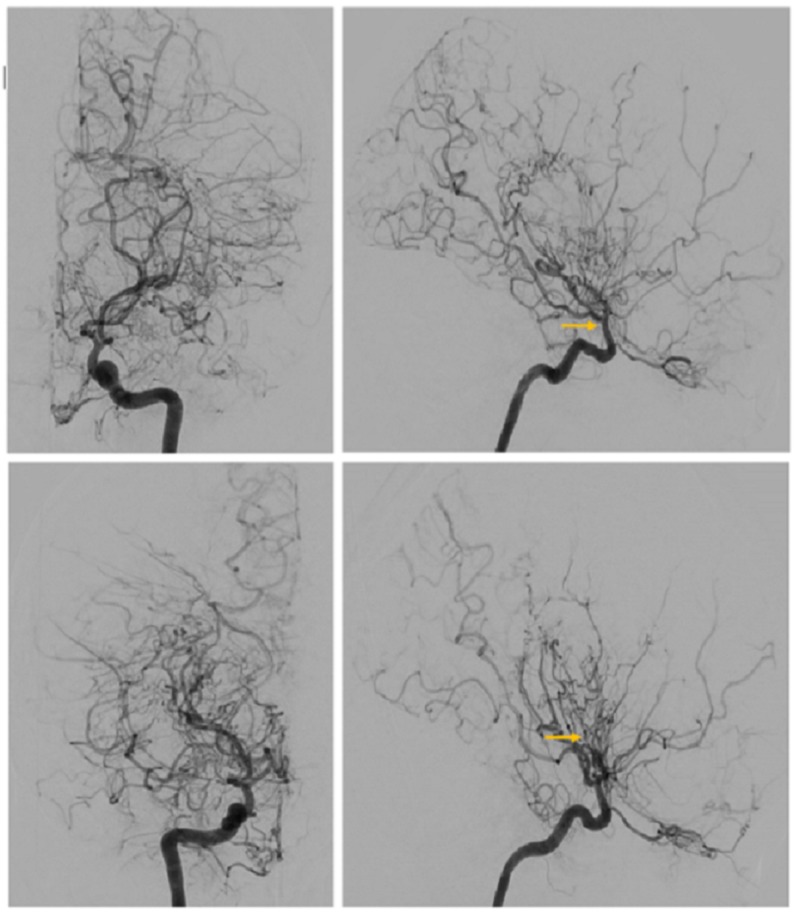
Top left (anteroposterior (AP) view) and top right (lateral view): left internal carotid artery angiogram showing stenosis of distal intracranial carotid artery at the supraclinoid segment (arrow). Bottom left (AP view) and bottom right (lateral view): right internal carotid artery angiogram showing a dense network of abnormally dilated collateral vessels with the appearance of a “puff of smoke” (arrow).

As shown in Figure 1, over 12 years ago, the patient was admitted for a left frontal hematoma that required an indirect revascularization of the left hemisphere through encephalo-duro-arterio-myo-synangiosis (EDAMS) to prevent further ischemia. Upon discharge, she was noted to have neurologic deficits, primarily expressive aphasia and right-sided weakness, although she was ambulating independently. Her family history was significant for hemorrhagic strokes on her paternal side of the family. A cerebral angiogram was performed to rule out arteriovenous malformations and aneurysms; however, a right-sided MMD pattern was observed (Figure 2).

Genetics was consulted, which led to the patient being found to have an RNF213 gene mutation. Due to the risk of hemorrhagic and ischemic stroke of the right hemisphere, the possibility of revascularization of the right hemisphere was discussed. However, she was not willing to consider treatment at the time. A psychiatric evaluation reported the patient to have expressive aphasia, unclear etiology of the patient’s catatonic state. Her catatonia included selective mutism, rigidity, immobility, fixed gazing, negativism, oppositional paratonia, and refusal for oral intake. Differential diagnoses of the aforementioned symptoms included apathetic delirium, depression, or a neurobiological disorder. She was initially treated with loxapine and mirtazapine, but yielded no improvement. Mild improvement occurred after changing loxapine to risperidone. A trial of 2 mg intravenous (IV) lorazepam (Ativan) briefly helped alleviate symptoms of catatonia, and the patient became less resistant to examination and paid more attention to her surroundings. However, she remained selectively mute. After two weeks with mirtazapine (15 mg), risperidone (1.5 mg), and clonazepam (1 mg bid), the patient showed notable improvement. She was more interactive with her surroundings, less agitated, cooperative to examination, able to follow simple commands, and able to get out of bed but remained non-verbal. Once clonazepam was decreased, she became less drowsy, able to eat independently and continued to show signs of further improvement. She became verbal, however, her speech was non-fluent, nonsensical, disorganized, and showed minimal language comprehension. The patient returned to being mute and refused to eat for the ensuing two weeks requiring nutrition to be maintained by a nasogastric tube. Mirtazapine was increased to 30 mg and risperidone to 2.5 mg. Repeated electroencephalogram (EEG) did not show epileptiform activity but showed slow waves consistent with structural changes. Electroconvulsive therapy (ECT) was briefly considered due to her continuing refusal to eat. Fortunately, the patient showed remarkable improvement. She began to eat, spoke clearly, and interacted with her family. Though she denied any depressive or psychotic features, she was observed smiling spontaneously for the first time since her admission. Major depression with catatonic features, complicated with comorbid medical conditions remained the preferred diagnosis.

## Discussion

The clinical presentation of MMD varies by age and includes focal neurological deficits, headaches, decreased level of consciousness, and neurocognitive impairment due to ischemic or hemorrhagic strokes [5]. In the pediatric population, MMD most commonly presents as transient ischemic accidents or as ischemic strokes, while in the adult population it is more likely to present as hemorrhagic strokes [3]. Even more so, in patients with MMD due to ischemia, kids are more likely to present with transient ischemic attacks and adults are more likely to present with ischemic strokes [1].

Surgical treatment consists of two methods of revascularization, namely, direct and indirect. Direct revascularization is the anastomosis of the most distal branch of the internal carotid artery (ICA) to a cortical artery of the middle cerebral artery. Indirect revascularization refers to vascularized tissue by the external carotid artery (dura, temporal muscle, or the superficial temporal artery itself) being placed adjacent to the brain in order to promote growth of new blood vessels [6].

Data on the cognitive sequelae of MMD is lacking, with very few cases published. It has been reported that chronic ischemia and concomitant significant brain hypo-perfusion can lead to cognitive impairment, intellectual decline, or mental retardation [7]. An examination of pre-surgical MMD patients reported neuropsychological injury in the spheres of intelligence, processing speed, executive functioning, and visual-spatial abilities [8]. On rare occasions, individuals can develop memory disturbance, irritability, or agitation, which can be misdiagnosed as a solo psychiatric disorder such as schizophrenia [9]. Schwartz et al. reported a patient with MMD who presented with mood disorder due to impaired anterior cerebral circulation [10]. Kim et al. also found that recurrent intracranial hemorrhages were a consequence for patients with MMD [11]. They also noticed that a recurrent hemorrhage would be more likely as the interval gets longer since the first hemorrhage [11]. Additional findings show that recurrent hemorrhages lead to an increase in neurological deficiencies with 100% neurological deficits seen after the fourth recurrent hemorrhage [11]. The catatonic presentation of this patient can be due to the cognitive impairments from her history of interventricular hemorrhages.

Currently, the relationship between MMD and catatonia or depression is not well understood. While depression or catatonia are typically considered psychiatric disorders, they can also present as complications of medical conditions. At present, even though the basis of catatonia is not completely understood, one theory posits catatonia to be a movement disorder, similar to Parkinson syndrome, as they share similar clinical presentation [12]. A review of the literature shows strong evidence of a relationship between MMD and depression or catatonia. Ghignone et al. reported an MMD patient who developed catatonia that was successfully treated using electroconvulsive therapy (ECT) [13]. Another study reported that 19 of 26 (73%) patients developed depression as a corollary of hemorrhagic MMD [14]. Another report of 30 patients with MMD showed that 37% of patients presented with significant emotional distress (depression and/or anxiety) and 23% with significant cognitive impairment (measured by test scores) [15].

A review of the previous literature provides further support for a vascular-cognitive model leading to depression, where depression is characterized as unbalanced inter-hemispheres, specifically, a hypoactive left hemisphere (LH) and a hyperactive right hemisphere (RH) [16]. Studies show that the RH is involved in selectively processing negative emotions and pessimistic thoughts [16]. Conversely, the LH is involved with the processing of pleasurable activities, and when hypoactive, leads to symptoms of anhedonia, which is a key feature of depression [16]. In the patient in the our case report, MMD was suspected to potentiate the risk and vulnerability for depression. Her mood may have made her avoid further treatment, which lead to a second hemorrhagic stroke and catatonia. This case highlights the complexity of the evolving neurosurgical and psychiatric complications of MMD.

At the heart of the matter, the question is whether the onset of depression after the LH stroke affected her decision to decline revascularization prior to her RH stroke. Most patients with moderate to severe major depression retain the capacity for personal and medical decision-making [17]. It is unclear if she would have required more formal competency evaluation and a matter of speculation whether more aggressive management of her depression could have altered her clinical trajectory. Some of the early warning signs of progressive depression in this patient included her lack of effort, not following up with her primary doctor, and non-compliance with the recommended antidepressant. It is a difficult balance of respecting a patient’s autonomy versus the realistic risk of additional bleeding. Her evolving depression most likely had some influence on her understanding of the risk and benefits associated with the treatment of her MMD. The psychiatric and neurological shifts of this patient’s baseline make it divisive as to consider her medical decision-making capacity as being sufficient. Càceda et al. state that depression and anhedonia, both of which this patient was diagnosed with, do not allow the patient to properly assess the benefits of their decisions [18]. They also pointed out that these patients have decreased activity in their ventral and dorsal striatum [18]. The ventral and dorsal striatum are linked to the middle cerebral artery, which is a branch of the ICA [19]. MMD is known to affect the ICA [20]. Therefore, if the patient’s decision-making capacity is compromised, it is questionable to trust the patient’s competency in order to determine their medical management.

In this case report, the patient did not want revascularization of the right hemisphere of the brain, which could lead to more grave outcomes, and so it is difficult to say if her decision was considered acceptable or if a healthcare proxy is more acceptable.

## Conclusions

MMD is a rare neurovascular disorder of unknown etiology. While this disease is complicated, it requires a holistic and interdepartmental approach for management. For our patient, neurosurgery, neurology, and psychiatry were consulted. While the medical complications of MMD have been well-documented, the psychiatric associations are unclear and lack information. Further research is needed to document the manifestation of psychiatric symptoms, as well as stratify cognitive symptoms according to disease severity and treatment. We also question if obtaining consent from a patient with an untreated hemorrhagic MMD is an appropriate course of action, and we hope that our encounter will guide healthcare providers strengthen patient-provider relationships, explore patient’s perspectives on treatments of MMD, and understand the potential outcome of untreated MMD. We hope that our case sheds light on the evolution of neuropsychiatric complications of MMD.
